# β-Barrels and Amyloids: Structural Transitions, Biological Functions, and Pathogenesis

**DOI:** 10.3390/ijms222111316

**Published:** 2021-10-20

**Authors:** Anna I. Sulatskaya, Anastasiia O. Kosolapova, Alexander G. Bobylev, Mikhail V. Belousov, Kirill S. Antonets, Maksim I. Sulatsky, Irina M. Kuznetsova, Konstantin K. Turoverov, Olesya V. Stepanenko, Anton A. Nizhnikov

**Affiliations:** 1 Laboratory for Proteomics of Supra-Organismal Systems, All-Russia Research Institute for Agricultural Microbiology, 3 Podbelskogo Sh., Pushkin, 196608 St. Petersburg, Russia; ansul@mail.ru (A.I.S.); a.kosolapova@arriam.ru (A.O.K.); belousovmix@gmail.com (M.V.B.); k.antonets@arriam.ru (K.S.A.); 2 Laboratory of Structural Dynamics, Stability and Folding of Proteins, Institute of Cytology, Russian Academy of Sciences, 4 Tikhoretsky Av., 194064 St. Petersburg, Russia; imk@incras.ru (I.M.K.); kkt@incras.ru (K.K.T.); lvs@incras.ru (O.V.S.); 3 Faculty of Biology, St. Petersburg State University, 7/9 Universitetskaya Emb., 199034 St. Petersburg, Russia; 4 Institute of Theoretical and Experimental Biophysics, Russian Academy of Sciences, 3 Institutskaya St., 142290 Moscow, Russia; bobylev1982@gmail.com; 5 Laboratory of Cell Morphology, Institute of Cytology, Russian Academy of Sciences, 4 Tikhoretsky Av., 194064 St. Petersburg, Russia; m_sulatsky@mail.ru

**Keywords:** amyloid, β-barrel proteins, amyloid fibrils, amyloidosis, amyloid aggregation, protein aggregation

## Abstract

Insoluble protein aggregates with fibrillar morphology called amyloids and β-barrel proteins both share a β-sheet-rich structure. Correctly folded β-barrel proteins can not only function in monomeric (dimeric) form, but also tend to interact with one another—followed, in several cases, by formation of higher order oligomers or even aggregates. In recent years, findings proving that β-barrel proteins can adopt cross-β amyloid folds have emerged. Different β-barrel proteins were shown to form amyloid fibrils in vitro. The formation of functional amyloids in vivo by β-barrel proteins for which the amyloid state is native was also discovered. In particular, several prokaryotic and eukaryotic proteins with β-barrel domains were demonstrated to form amyloids in vivo, where they participate in interspecies interactions and nutrient storage, respectively. According to recent observations, despite the variety of primary structures of amyloid-forming proteins, most of them can adopt a conformational state with the β-barrel topology. This state can be intermediate on the pathway of fibrillogenesis (“on-pathway state”), or can be formed as a result of an alternative assembly of partially unfolded monomers (“off-pathway state”). The β-barrel oligomers formed by amyloid proteins possess toxicity, and are likely to be involved in the development of amyloidoses, thus representing promising targets for potential therapy of these incurable diseases. Considering rapidly growing discoveries of the amyloid-forming β-barrels, we may suggest that their real number and diversity of functions are significantly higher than identified to date, and represent only “the tip of the iceberg”. Here, we summarize the data on the amyloid-forming β-barrel proteins, their physicochemical properties, and their biological functions, and discuss probable means and consequences of the amyloidogenesis of these proteins, along with structural relationships between these two widespread types of β-folds.

## 1. Introduction

Amyloids are insoluble fibrillar protein aggregates that are rich in β-sheets and possess a unique spatial structure called “cross-β” [1]. It was found that during the formation of an amyloid fibril, β-strands of the protein monomers form intermolecular β-sheets perpendicular to the fibril axis [2,3,4]. Stacks of β-sheets form an amyloid protofilament, which is the main subunit of a mature fibril composed of intertwined protofilaments [5]. This type of fold is named after its “cross-β” diffraction pattern with ~4.7 Å and ~10 Å scattering diffraction signals, referring to the interstrand distance and the distance between β-sheets, respectively [4,6]. The cross-β structure is the basis of an amyloid’s physicochemical properties, such as resistance to proteases [7] and ionic detergents [8], as well as its ability to specifically interact with Congo red (CR) and Thioflavin T (ThT) dyes, causing “apple-green” birefringence in polarized light upon CR binding [9,10], and increasing the fluorescence intensity of the fibril-bound ThT [11]. Amyloids are best known for their association with the development of incurable disorders called amyloidoses, such as Alzheimer’s disease and type II diabetes mellitus [12,13]. At the same time, amyloids can perform various physiological functions [14,15]. The number of discovered “functional” amyloids is growing rapidly and, to date, such amyloids have been identified within diverse groups of organisms, including bacteria, archaea, fungi, animals, and plants [16,17,18].

The ability of proteins to form amyloids is determined by the presence of specific aggregation-prone regions in their amino acid sequence. These regions are called amyloidogenic, and can be predicted by different bioinformatic approaches [19]. These regions differ in their structure and amino acid composition, but there are at least two well-defined groups of such regions: (1) Q and/or N-rich, which are typical for infectious amyloids of yeast and several pathological amyloids of mammals [20], and (2) rich in hydrophobic residues, which are found in various pathological and functional amyloids. Not only composition, but also the position of a particular residue, is highly important for amyloidogenesis driven by the type II amyloidogenic regions [21]. The presence of predicted amyloidogenic regions in a protein does not define its capability to form amyloids in vivo—which, in fact, depends on a number of factors (level of expression, protein quality control systems, posttranslational modifications, etc.) [22,23].

Although the association of amyloid-forming properties with protein domains is poorly studied, there is growing evidence that different functional and pathogenic amyloid-forming proteins of prokaryotes and eukaryotes contain β-barrel domains or form β-barrels under particular conditions in vitro or in vivo. Similarly to amyloids, β-barrels represent a highly specific type of β-strand-enriched fold [17,24,25,26], which is widespread in nature and is presented by both membrane and cytoplasmic proteins [27]. Comparably to amyloidogenic proteins, β-barrel proteins are rich in hydrophobic residues [28]. β-barrels are composed of β-strands connected by loops. β-strands have a predominantly antiparallel orientation and are hydrogen-bonded to one another, which leads to the formation of β-sheets. When the first and last β-strands interact with a hydrogen bond, β-sheets are twisted into a cylindrical structure, resembling a barrel, from which the name of this type of fold is derived [25,27,29,30]. The similar topology of β-barrel proteins may indicate their common evolutionary origin. Despite some structural similarities, β-barrel proteins can perform a wide range of biological functions, including transporting and signaling, acting as storage proteins, and many others [25,29,30,31,32]. The relationship between β-barrels and amyloid folds is of great interest.

In this review, we analyze rapidly accumulating data on the involvement of β-barrel proteins of prokaryotes and eukaryotes in amyloid fibrillation, observe probable mechanisms of the formation of amyloids by β-barrel proteins, and vice versa: the formation of β-barrels at intermediate steps of amyloidogenesis. Finally, we discuss the functional and pathological roles of these biological processes.

## 2. Aggregation-Prone β-Barrel Proteins: Structure, Diversity, and Biological Roles

Despite the variety of structures and functions of β-barrel proteins, they can be divided into two major groups: (1) water-soluble proteins of prokaryotes and eukaryotes, and (2) membrane proteins that are found in the outer membranes of Gram-negative bacteria, mitochondria, and chloroplasts [33,34], and produced by several species of Gram-positive bacteria [35]. The main structural difference between water-soluble and membrane β-barrels is the orientation of their nonpolar and polar amino acid residues. In the case of water-soluble β-barrel proteins, hydrophobic residues are oriented inside the cylinder, resulting in the formation of a hydrophobic core, while polar residues are on the surface of the barrel, being solvent-exposed. In contrast, in membrane β-barrel proteins, hydrophobic residues are oriented outward, and interact with the surrounding lipids, while their hydrophilic residues are fed to the inside of the barrel and form a pore. Membrane β-barrels can act as membrane channels specific to certain types of molecules (porins), and participate in the transport of proteins. Currently, approaches to the computational design of transmembrane β-barrels are being developed that have potential both for understanding the determinants of these proteins’ folding and membrane insertion, and for the custom engineering of nanopores formed by them [36].

Soluble β-barrels can have a chromophore that determines their optical properties. They can bind and transport small hydrophobic molecules with high affinity (lipocalins), bind the superoxide radical anion in the active center, activate cell surface receptors, participate in protein storage defense from pathogens, and perform other functions [37,38,39,40,41,42,43].

**Figure 1:**
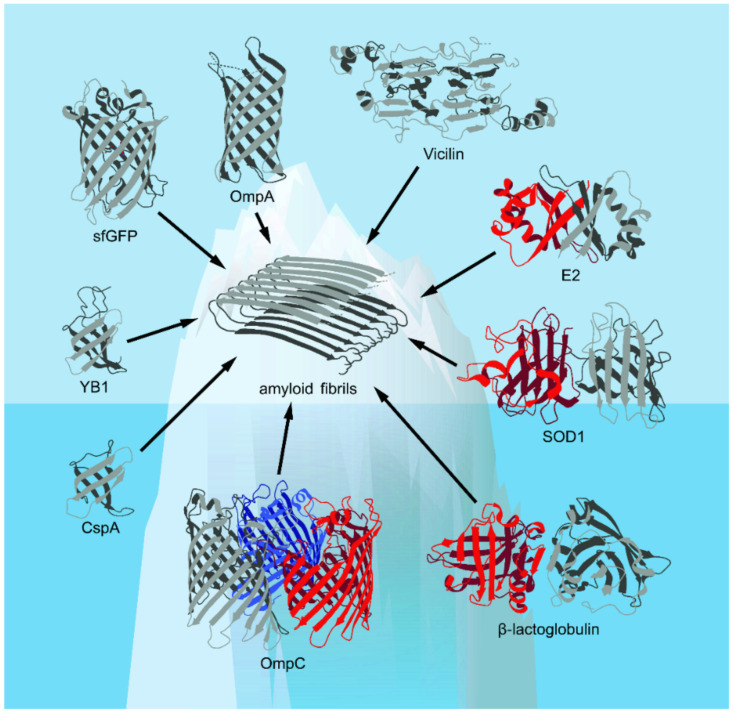
The spatial structures of the β-barrel proteins discussed in this review presented in the Protein Data Bank (PDB) database (Available online: https://www.rcsb.org/ (accessed on 17 October 2021))—with the exception of vicilin, whose structure is computationally predicted (based on [18], with modifications)—are shown. Corresponding elements of the secondary structure, including α-helices and β-strands, are shown. The figure shows that known examples of amyloids formed from β-barrel proteins are likely to represent only “the tip of the iceberg” of their real number.

Here, we will consider in detail the structural features and biological functions of only those proteins with β-barrel domains—which, according to the literature, are capable of forming amyloid or amyloid-like fibrils in vitro and/or in vivo (the amyloid-forming β-barrel proteins, for which experimentally confirmed structural data are available, are shown in Figure 1). The structure and functions of these proteins in the monomeric β-barrel state are discussed in this section. The mechanisms and functional consequences of aggregation of β-barrels and their amyloid formation are discussed in Section 2 and Section 3, respectively.

### 2.1. DNA-Binding Proteins of Viruses

The rather unusual eight-stranded β-barrels of the DNA-binding proteins EBNA1 of the Epstein–Barr virus and E2 of the papillomavirus HPV16 are generated via dimerization: the barrel contains four β-strands from each monomer [44,45]. The α-helical segments located on both sides of the β-barrel of these viral proteins are necessary for interaction with DNA. The HPV16 papillomavirus E2 β-barrel is engaged in the regulation of transcription and viral DNA replication. EBNA1 plays a key role in Epstein–Barr virus episome replication and maintenance of their presence in cells during the latent phase of infection [46]. E2 is capable of forming amyloid-like fibrils in vitro [47], while EBNA1 forms spherical oligomers possessing several properties of amyloids [48].

### 2.2. Outer Membrane Proteins of Gram-Negative Bacteria

Outer membrane proteins (OMPs) are a group of proteins from Gram-negative bacteria, chloroplasts, and mitochondria [37]. OMPs of bacteria represent β-barrel proteins, which comprise 6–26 antiparallel β-strands incorporated into the outer membrane of Gram-negative bacteria [49]. OMPs can act as passive transport pores [50,51] and enzymes [52], contribute to membrane stability and integrity [53], and promote iron uptake [54,55] and drug resistance [56,57]. In pathogenic bacteria, OMPs promote adhesion and invasion [58,59,60]. Recent findings demonstrate that several OMPs are amyloidogenic [61,62,63,64] (see Section 3.2. for a detailed description of the OMP amyloid formation).

OMPs are mostly studied with the usage of *Escherichia coli* as a model organism. OmpA from *E. coli* is one of the most abundant proteins of the outer membrane, with approximately 100,000 copies per cell [65]. The relatively small size of that protein’s monomer has made it a popular model for the study of transmembrane β-barrel protein folding. The N-terminal domain of OmpA folds into eight transmembrane β-strands with three short periplasmic loops and four longer surface loops, whereas the C-terminal domain is globular and located in periplasmic space, where it is likely to interact with the peptidoglycan layer and periplasmic proteins [66,67,68]. It has been also suggested that OmpA can alternatively adopt 16 β-strand conformation with the formation of an additional 8 β-strands by the C-terminal domain [69]. OmpA is synthesized as proOmpA with an N-terminal signal peptide [70]. The proOmpA polypeptide is transported into periplasmic space through the SEC pathway, with concurrent removal of the signal peptide. Incorporation into the outer membrane and folding of the OmpA protein are completed via the BAM complex [49]. OmpA is known for its role in the maintenance of membrane integrity and stability, biofilm formation, adhesion, and invasion [71].

OMPs can also assemble into trimeric pore channels; that functional group of OMPs includes general porins (for example, OmpF, OmpC, and PhoE from *E. coli*) and substrate-specific porins, including the maltooligosaccharide-specific maltoporin LamB from *E. coli* [65].

Outer membrane phospholipase A (OMPLA) of *E. coli* is an example of an OMP with enzymatic activity. Folded phospholipase A includes 12 transmembrane β-strands [72]. The enzyme is active in a dimeric form, and Ca^2+^ is required as a cofactor [72,73]. OMPLA catalyzes the hydrolysis of membrane phospholipids by removing the ester bond from the glycerophosphodiester backbone [52]. OMPLA also contributes to the integrity and stability of the outer membrane [74].

### 2.3. Proteins Containing the Cold-Shock Domain

Another representative of amyloidogenic β-barrels is the cold-shock domain (CSD), which is present in bacterial cold-shock proteins (CSPs) and Y-box proteins of eukaryotes. This domain consists of five antiparallel β-strands folded into a barrel [38]. Proteins containing the CSD bind single-stranded nucleic acids [38], thus acting as mRNA chaperones and participating in the regulation of transcription and translation [75,76]. The highly structured CSD domain of eukaryotic Y-box proteins is flanked by extended disordered regions at the N- and C-termini, which probably determines the ability of these proteins to be incorporated into numerous cytoplasmic and nuclear membraneless organelles [77,78,79,80]; these proteins can also bind to fibrillar structures in a cell [81,82]. This expands the role of eukaryotic Y-box proteins in the regulation of a wide variety of processes, including RNA splicing, DNA reparation, and replication, and suggests their involvement in the stress responses, embryonic development, differentiation, oncogenic cell transformation, and metastasis [83].

### 2.4. Cupins

The cupin superfamily is one of the largest groups of proteins. Cupins are found within eukaryotes, bacteria, archaea, and viruses [39]. The distinguishing feature of these proteins is the presence of one or several cupin domains (after the Latin “cupa”—small barrel) [84]. The cupin domain is a β-barrel domain that includes two conservative β-strand motifs separated by an intermotif region [84,85]. Proteins from the cupin superfamily usually possess one (monocupins) or two cupin domains (bicupins) [85]. Most monocupins and bicupins are prone to oligomerization [86].

Proteins of the cupin superfamily are extremely diverse in function. To date, the cupin superfamily consists of thousands of proteins from no less than 50 protein families [87,88]; it comprises a wide variety of metal-binding enzymes, including dioxygenases, isomerases, hydrolases, and non-enzymatic proteins such as seed storage globulins and sugar-binding proteins [89].

The study of the cupin superfamily started with the identification of the conserved motif shared by germin and germin-like proteins of plants, and spherulin of the slime mold *Physarum polycephalum* [84]. Germin is a wheat oxalate oxidase found in germinating wheat embryos, which contributes to seed development and pathogen defense [90,91]. Germin is an oligomeric monocupin that forms homohexamers with extreme resistance to dehydration, proteases, and heat [92].

Seed storage globulins were the first discovered bicupin proteins of the cupin superfamily [93]. This group includes legumins (hexameric 11S globulins) and vicilins (trimeric 7S globulins) [94]. The *Pisum sativum* L. vicilin has recently been shown to form functional amyloids in plant seeds [18]. Apart from their function as storage proteins, seed storage globulins are implicated in the defense of plants from pathogens, and represent the major allergens from legumes [95,96,97,98].

### 2.5. GFP-like Proteins

GFP-like proteins (named after avGFP—the first green fluorescent protein found in the jellyfish *Aequorea victoria*, [40])—including fluorescent proteins (FPs) and non-fluorescent chromoproteins—can be regarded as the best-known proteins with a pronounced β-sheet structure. The barrel of GFP-like proteins is composed of 11 β-strands wrapped around a central α-helix containing a chromophore synthesized as a result of posttranslational modification of three of its amino acid residues [99]. The barrel of GFP-like proteins serves as a platform for the assembly around the chromophore-forming tripeptide of catalytic amino acids and the amino acids that regulate the photophysical behavior of the protein [100,101,102,103,104].

The biological functions of GFP-like proteins, despite numerous assumptions, are not completely clear, and are not always determined by the ability of these proteins to exhibit fluorescence [105,106]. It is assumed that different types of coloration (bioluminescent/fluorescent in jellyfish, and fluorescent/non-fluorescent in corals) are involved in the visual predator–prey communication [107], intraspecies recognition [108,109], and camouflage or attraction of symbiotic algae [110,111]. Re-emission and scattering of light—mainly by blue and green fluorescent proteins—provides photoprotection of symbionts [111,112], and prevents overheating of deep coral tissues [113]. It is assumed that green FPs may be involved in the antioxidant defense of corals [114]. Several possible functions of green FPs—for example, the regeneration of coelenterazine in bioluminescent organisms—are associated with their ability to act as electron donors for biologically significant oxidants [115]. It should be noted that the unique ability of GFP-like proteins to fluoresce in the visible region of the spectrum determines the wide application of fluorescent biomarkers and biosensors developed on their basis for solving numerous fundamental and practical problems of cell biology and medicine via fluorescent methods [116,117,118,119].

### 2.6. Other Amyloidogenic Eukaryotic β-Barrel Proteins

The enzyme superoxide dismutase-1 (SOD1) is a dimer of two β-barrels composed of eight antiparallel strands, the structure of which is stabilized by the binding of Zn and Cu ions. SOD1 also utilizes Cu ions to catalyze the conversion of superoxide to oxygen and hydrogen peroxide. SOD1 is considered to be a crucial player in the antioxidant defense of cells that are in any way in contact with oxygen [42].

Acidic fibroblast growth factor (FGF-1) consists of 12 β-strands that are arranged in 3 similar lobes around the central axis, with 6 strands forming an antiparallel [120,121]. This protein plays a key role in the processes of proliferation and differentiation of a wide range of cells and tissues [43].

β-lactoglobulin is the second most abundant protein in the milk of different mammalian species, excluding humans and rodents; it has a compact structure including a β-barrel consisting of nine β-strands, a hydrophobic region, and one α-helix, and forms homodimers under physiological conditions [122]; its functions remain unclear, but β-lactoglobulin has potential industrial applications due to its ability to aggregate and to form gels and stable foams [123].

Thus, the localization, unique properties, and diverse structural features of β-barrel proteins (in particular, the smallest known β-barrels have only 6 β-strands, while the largest have 26 β-strands [124]) determine a wide range of their biological functions (transport, signaling, protection, storage, binding, regulatory, etc.) [25,31,32]. Numerous studies aimed at elucidating the structural features of β-barrel proteins have led to the conclusion that correctly folded β-barrels not only function in the monomeric (dimeric) form, but also tend to interact with one another, followed by the formation of higher order oligomers or even aggregates. Next, we will consider the prerequisites for the aggregation of β-barrels during the folding process, and the aggregation associated with degradation of the native proteins. We will also observe various types of formed aggregates and their possible biological roles.

## 3. Folding of β-Barrel Proteins and Their Aggregation

### 3.1. Particularities of the β-Barrels’ Folding

Protein folding is the acquisition of a unique native spatial structure by a polypeptide chain. The transition of a protein from a fully expanded state to a native state can be described by a free-energy landscape, i.e., the dependence of the free energy of the protein on all coordinates that determine the state of the system [125,126,127]. Within the framework of this model, the unfolded state of the polypeptide chain corresponds to a wide “hilly plateau” of free energy, which indicates the possibility of realizing this state via a large number of different conformations of the main chain. The number of these conformations decreases when approaching the native state; therefore, the energy surface illustrating the process of protein folding is called the “energy funnel” [125,126,127].

Due to the unique topology of the membrane β-barrels, their folding is a rather complex and prolonged process, which is associated with the accumulation of a number of intermediate states [128]. In the case of the bacterial outer membrane β-barrels, productive folding in vivo employs ATP-dependent cellular machinery: chaperones and proteins with chaperone activity—which provide subcellular delivery of unfolded β-barrel proteins, promote their correct folding, and prevent aggregation—and the barrel assembly machinery (BAM) complex, which is responsible for the packaging and localization of transmembrane β-barrels in the membrane [129,130]. The central subunit of the BAM machine, BamA, has the β-barrel domain that provides the integration of different partially folded β-barrel proteins into membranes via the so-called “swing” mechanism [131]. Intermolecular interactions between the correctly folded polypeptide chains of β-barrels in some cases lead to their oligomerization (the formation of their quaternary structure).

The folding of soluble β-barrels, which are the “inside-out” version of membrane β-barrels (since the inward regions of the former exhibit similar hydrophobicity with the outward regions of the latter, and vice versa [28]), may not require special folding machinery, and may occur in an ATP-independent manner, as in the case of GFP-like proteins. Moreover, co-translational folding of GFP occurs more efficiently than after its chemical denaturation, which can be partially explained by the proposition that gradual polypeptide synthesis occurring in ribosomes facilitates the acquisition of more favorable conformations for correct folding [132]. Several soluble β-barrels, such as human SOD1, require special chaperones (hCCS in the case of SOD1) for correct folding and maturation, which occur through a series of consequent intermediate steps [133]. The misfolded aggregated state of SOD1 has also been shown to interact with molecular chaperones [134,135].

### 3.2. Formation of the Quaternary Structure of β-Barrels as a Result of Their Oligomerization

Most of the known GFP-like proteins—with exception of specially designed, highly soluble variants [136]—are prone to oligomerization and aggregation; they form either dimers [137], tetramers [108,138,139], or high-molecular-weight oligomeric complexes [140,141]. Different variants of GFP-like proteins developed as a result of structure-guided rational design, which have been characterized as monomers in vitro, can oligomerize in the cell [142]. When expressed in mammalian cells, GFP-like proteins can form fluorescent crystals ~4–7 µm in size, as was shown for the photoconvertible protein KikGR [143]. GFP-like proteins can display similar properties in nature: green FP forms spindle-like aggregates and diamond-shaped crystals ~5–10 µm in size in the tissues of the *Zoanthus sp*. polyp [144].

EBNA1 dimers can form oligomers through interactions between alternative protein regions. For example, the DNA-binding region of EBNA1 in the absence of DNA crystallizes in the form of a hexameric ring formed by three dimeric β-barrels [145], while in the presence of DNA, it crystallizes in the form of a tetramer (dimer of dimeric β-barrels), with a different morphology [146]. It is assumed that the interaction of EBNA1 in different oligomeric states with different regions of DNA is associated with the multiple functions of this protein. Tetrameric [147] and hexameric [148] oligomers with a different spatial organization than the EBNA1 hexamers are found upon crystallization of the DNA-binding site of free E2. It was hypothesized that the oligomerization of E2 plays a significant role in the spatial organization of the viral DNA region *ori* [149].

Most bacterial CSPs are considered monomers, but some representatives of this class are capable of forming dimers of various morphologies in solution and during crystallization [150,151,152], including domain-swapped dimers [153,154]. Eukaryotic YB-1 and its homologs are prone to aggregation with the formation of high-molecular-weight complexes (up to 800 kDa), presumably due to the interaction of disordered C-terminal regions [155,156]. Intracellular SOD isoforms have a dimeric (SOD1, [157]) or tetrameric (SOD2, [157]) organization, while extracellular SOD3, being a tetramer, is capable of forming octamers and higher order oligomers [158,159,160].

**Figure 2:**
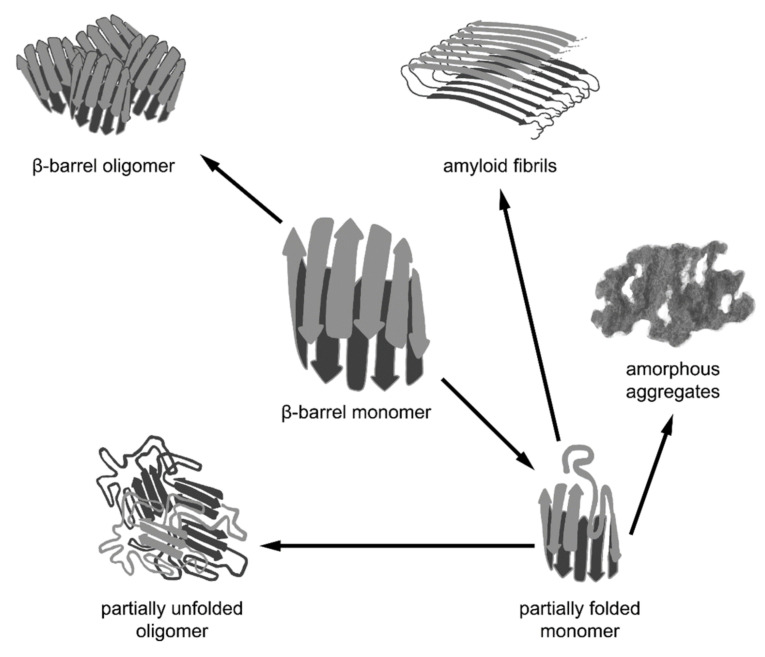
Different pathways of aggregation of β-barrel proteins. β-barrel proteins are prone to associating and aggregating. The formation of functional oligomeric complexes of native proteins is caused by intermolecular interactions between the correctly folded polypeptide chains of β-barrels. However, β-barrel proteins can aggregate not only in their native state via the interaction of correctly folded monomers, but also as a result of the misfolding or distortion of their native structure, leading to the formation of non-native oligomers, amorphous aggregates, and amyloid fibrils. It should be noted that amyloid fibrils formed by some β-barrel proteins may represent a functional native state.

Thus, various cellular cofactors are exploited in the complex multistage process of β-barrel folding, which facilitates the transition of the unfolded β-barrel polypeptide chain into a monomeric (in some cases, oligomeric) native structure. However, β-barrels can often form higher order aggregates with different properties. The formation of functional complexes of native proteins (tetramers, octamers, hexamers forming spindle-like and annular aggregates, rhomboid crystals, etc.) is caused by intermolecular interactions between the correctly folded polypeptide chains of β-barrels. Nevertheless, β-barrel proteins can aggregate not only in the native state via the interaction of correctly folded monomers, but also as a result of the misfolding or distortion of their native structure, leading to amyloid formation. Another possible variant is the formation of functional amyloids in vivo by the β-barrel proteins for which the amyloid state is native (Figure 2).

## 4. The Transition of β-Barrel Proteins to Amyloids

### 4.1. Misfolding of Proteins: Pathological and Functional Amyloids

The transition of a protein from an unfolded to a unique native state can follow different routes, and can be accompanied by the formation of stable steady-state or kinetic intermediates. Some of these intermediates can occur along the folding pathway—i.e., contain structure elements present in the native state (“on-pathway”)—while others accumulate outside this route (“off-pathway”) [161,162,163]. The appearance of folding intermediates with a non-native structure may be induced by mutations in a protein’s amino acid sequence, or by impairment of the normal folding process by external factors. In addition, such intermediate states can be populated via denaturation of the native protein structure as a result of external influences. With the accumulation of a large number of polypeptide chains in an incorrectly folded state (in the case of abnormal protein folding or denaturation of the native protein [164,165]), when cellular machinery cannot cope with the restoration of the native protein structure or degradation of the incorrectly folded proteins, the appearance of their intermolecular contacts and aggregation can occur.

For a long time, it was believed that the transition between the native and denatured states of the protein is reversible, and the formation of aggregates is an artefact [166]. In this regard, the processes of protein aggregation during folding had not received adequate attention. The situation changed markedly when the link between the accumulation of amyloid fibrils and various, mostly incurable diseases called amyloidoses—associated with violation of protein folding—became obvious. Amyloidoses are characterized by the pathologic accumulation of extracellular or intracellular protein deposits in the fibrillar amyloid state [12]. To date, ~50 different pathological amyloids have been identified in vertebrates—mostly humans [12]—with an even greater number of diseases (aggregation of the same protein can lead to several pathologies) [13,167,168]. Amyloidoses may have different origins (primary or secondary; systemic or localized; acquired or inherited). Different neurodegenerative diseases—such as Alzheimer’s disease [169], Creutzfeldt–Jakob disease [170], Parkinson’s disease [171], and others—are also associated with amyloid formation. Several tumors are accompanied by the formation of mutant p53 amyloids [172,173]. Still, any causal relationship between amyloid formation and disease development in such cases—as well as in the cases of several other amyloid diseases, such as type II diabetes mellitus [174]—remains unclear.

It should be noted that although the term “amyloid” has for a long time been mainly attributed to pathological processes and diseases, recent studies indicate that proteins in the amyloid state can also perform essential physiological functions [16,175]. Such “functional” amyloids have been found in all three domains of life. These protein aggregates perform various functions, including mechanical protection and modification of the cell surface, biotic or abiotic surface adhesion and invasiveness, internalization of cells, ensuring resistance to various antimicrobial agents, biosynthesis of pigments, homeostasis control, storage and release of hormones, storage of nutrients, signal transduction, etc. [176,177,178]. Note that the amyloid state is not a result of misfolding but, rather, a native conformational state for functional amyloids. Moreover, the formation of several functional amyloids is precisely controlled by cellular machinery including specific folding systems [179,180,181].

Some of the currently known amyloids of eukaryotes and prokaryotes—both pathological amyloids, and those performing physiological functions in various species—are formed by proteins sharing a similar β-barrel structure. In addition, the amyloidogenic properties of some β-barrels have been shown in vitro. Next, we will discuss amyloids of proteins with the β-barrel structure, and the factors contributing to their formation.

### 4.2. Diversity and Possible Biological Roles of Amyloids Formed by β-Barrel Proteins

#### 4.2.1. Amyloid Formation from β-Barrel Proteins of Viruses

It was found that the DNA-binding protein E2 of papillomavirus HPV16, which has a native β-barrel structure, is capable of fibrillogenesis in vitro in the presence of 2,2,2-trifluoroethanol (TFE) at a low concentration [47]. The formation of a molten globule-like state with a pronounced β-structure is a prerequisite for the formation of amyloid fibrils by the E2 protein of papillomavirus HPV16 [47]. The ability of this protein to form amyloids was assigned to the dynamic structure of the DNA-binding segment and the existence under native conditions of equilibrium between two protein conformations, with the DNA-binding site either adopting an α-helix fold or being partially denatured. Presumably, the fibrillogenesis of the E2 protein is associated with the refolding of the DNA-binding site into a non-native β-structure, and subsequent oligomerization and protein aggregation. Due to the marginal stability of the DNA-binding region of the E2 protein, the possibility of its fibrillogenesis in the cell cannot be ruled out. It is likely that such amyloids may contribute to the modulation of the DNA-binding activity of this protein.

Another viral protein—EBNA1 of the Epstein–Barr virus—is capable of forming only amyloid-like oligomers in vitro [48]. This is likely due to the significantly higher resistance of EBNA1 to external influences compared to E2. It is noteworthy that the formation of amyloid-like oligomers by the EBNA1 protein is also associated with the refolding of the DNA-binding region of the protein and the accumulation of a molted globule-like state with a pronounced β-structure, but requires preliminary complete unfolding of the protein [48]. It was suggested that EBNA1 expressed at a high level could accumulate in the cell in an oligomeric state with a pronounced β-structure, because the intracellular folding machinery is unable to ensure the folding of the protein into the native dimer performing certain specific functions—probably related to the modulation of DNA replication and episome segregation efficiency.

#### 4.2.2. Amyloid Formation by β-Barrel Proteins of Prokaryotes

Amyloid-like properties of the OmpA [59] and OmpC [62] proteins of *E. coli* and the Omp2-like protein [63] of *Mannheimia haemolytica*, belonging to the outer membrane β-barrel porins, were demonstrated in vitro. Outer membrane proteins are known to be involved in host–pathogen interactions and virulence [58,182]. The intraperitoneal injection of OmpC purified from *E. coli* cells was found to cause neurodegeneration in mice via calcium-dependent apoptosis [62]. For the Omp2-like protein, the presence of its fibrillar state in the biofilms of *M. haemolytica*, along with effects on the adherence of the bacterium to mammalian cells, were demonstrated [63]. Thus, we can hypothesize that at least several outer membrane porins of Gram-negative bacteria could exist in vivo in two different conformational states: transmembrane β-barrel, and amyloid. Mechanisms of amyloid formation by outer membrane porins in vivo are currently unclear, as are their biological functions. Based on the known functions of porins and the aforementioned studies, we may suggest that the amyloid states of the outer membrane proteins could contribute to biofilm formation and cell adherence, thus modulating the efficiency of host–pathogen interactions.

A proteomic screening and identification of amyloids assay (PSIA) [183,184] was used in our work to identify potentially amyloidogenic proteins in the proteome of the root nodule bacterium *Rhizobium leguminosarum* [64]. Out of 54 identified proteins, 2 proteins—RopA and RopB—were selected, as they have bioinformatically predicted β-barrel structures, and are probably involved in the control of plant–microbial symbiosis. It was shown that full-length RopA and RopB form amyloid fibrils in vitro, and that heterologically expressed RopA and RopB can aggregate in yeast and form amyloid fibrils on the surface of *E. coli*. Most importantly, it was confirmed that amyloid fibrils are formed from RopA and RopB directly in capsules of *R. leguminosarum* in vivo [64]. Our data suggest that RopA and RopB are functional amyloids, most likely involved in the symbiont–host supra-organismal interactions, although it remains unclear whether in vivo they form both transmembrane β-barrels and amyloids, or are present in the amyloid state only.

It was shown that cold-shock protein CspA from *E. coli* also forms amyloid-like fibrillar structures in vitro. Its CSD domain with a β-barrel structure is responsible for the protein’s capacity for fibrillogenesis. The formation of amyloid-like Ec-CspA fibrils under acidic conditions is associated with protein denaturation and the formation of an intermediate state in which a fragment of β-strands 1–3 is structured [185].

Thus, different β-barrel proteins of bacteria form amyloid fibrils in vitro, while some of them are involved in host–pathogen and host–symbiont interactions, and form functional amyloids in vivo.

#### 4.2.3. Amyloid Formation by β-Barrel Proteins of Eukaryotes

Recently, we have shown that the seeds of the garden pea *P. sativum* L. contain amyloid aggregates of the 7S globulin vicilin [18]. Using a wide range of physicochemical approaches, we demonstrated that vicilin forms amyloids in vivo and in vitro. Full-length vicilin contains two evolutionarily conserved β-barrel domains—cupin-1.1 and cupin-1.2—which form amyloid fibrils in vitro with physicochemical properties similar to those formed by the full-length vicilin. Interestingly, fibrils formed from cupin-1.2, in contrast to fibrils formed from cupin-1.1, can serve as seeds for the fibrillogenesis of vicilin, suggesting different involvement of these domains in the formation of amyloids by the full-length protein. In vivo, vicilin forms amyloids in pea cotyledon cells. The amount of vicilin amyloids increases during seed maturation, and drastically decreases during germination, suggesting the presence of amyloid-disassembling machinery that likely acts as a chaperone and/or protease in seeds. This made it possible for us to assume the essential role of amyloid formation in protein storage in plant seeds. It is likely that amyloid formation protects storage proteins from degradation and enables the long-term survival of plant seeds. In addition, it was shown that vicilin amyloids exhibit toxicity to yeast and mammalian cells, which may indicate their involvement in the defense from pathogens (in particular, fungicidal activity) [18]. The vicilin amyloid represents the first identified functional amyloid formed by a β-barrel protein of eukaryotes. Other examples of amyloids formed by the β-barrel proteins are presented either by pathogenic amyloids or by proteins whose amyloidogenesis was demonstrated in vitro only.

The formation of amyloid fibrils from native monomeric newt fibroblast growth factor nFGF-1 in vitro was shown in the presence of TFE (as in the case of DNA-binding proteins) [186]. The first conformational transition observed for nFGF-1 in the presence of TFE at concentrations above 10% (*v*/*v*) is associated with the destruction of hydrophobic contacts that stabilize the native structure of the β-barrel. This leads to the formation and accumulation of a partially folded intermediate state with extended β-sheets and loosely packed side chains. In this state, the protein has a high tendency to aggregate due to the hydrophobic surface being exposed to the solvent. Protein aggregation and the formation of amyloid fibrils were observed during prolonged incubation (> 3 h) of the protein in TFE in the concentration range from 10 to 40% (*v*/*v*). Based on the obtained data, it was assumed that the formation of amyloid-like fibrils from nFGF-1 occurred due to the rearrangement of extended β-sheets and the formation of new intermolecular hydrogen bonds. Exposure to the solvent of nonpolar amino acid side chains of the protein in the intermediate non-native conformation also stimulates the aggregation of this protein [186]. The formation of amyloid fibrils by nFGF-1 was shown in vitro only, and is unlikely to occur in vivo, where it might instead cause the loss of function of this protein.

The formation of amyloid fibrils in vitro under the influence of external factors (temperature and low acidity) was also shown for the superfolder GFP (sfGFP) with a native β-barrel structure [187]. The unfolding/refolding of GFP-like proteins induced by various chemical denaturants and heating is complicated by the formation of several intermediate partially folded states [188,189,190,191,192,193,194]. It was shown that the intermediate states formed during thermal denaturation of the GFP-cycle3 retained extended structured regions of several β-strands [195]. One of these states revealed during the unfolding of GFP upon heating, and under the action of chemical denaturants, is characterized as a molten globule-like state [189,194,195,196]. The exposure of clusters of hydrophobic amino acids of GFP in an intermediate state is considered to be the main reason for abnormal protein aggregation upon heating [197]. Interestingly, amyloids formed from sfGFP in vitro lose their green fluorescence, and are toxic to mammalian cells [187]. It is unclear whether GFP-like proteins can form bona fide amyloid fibrils in vivo but, as was mentioned above, they show high oligomerization and, in several cases, aggregation propensity. They may also specifically bind amyloid aggregates of other proteins in a sequence-independent manner, inhibiting their fibrillation, as was recently shown [198].

Aggregation and accumulation of the fibrillar inclusions of SOD1, consisting of two β-barrels in the native state, occur during the development of sporadic and hereditary forms of human amyotrophic lateral sclerosis (ALS). Though the development of ALS can be accompanied by the aggregation of different proteins, SOD1 aggregation is considered to be an important factor of this disease, and could lead to toxicity, causing motor neuron death [199,200,201]. It has been proposed that the inclusion of SOD1 in aggregates is promoted by partial denaturation of the enzyme [202], which may occur due to its destabilization by the mutations in its structural gene [203], and by binding of Ca^2+^ ions [204], or as a result of low stability of a newly synthesized enzyme in the apoform, which is not yet complexed with metal ions [205,206]. It should be mentioned that although SOD1 forms amyloid-like aggregates in vitro [200] and in transgenic mice [207,208], it is unclear whether the SOD1 aggregates identified in tissues of patients with ALS are bona fide amyloids.

Human YB-1 protein is also capable of forming amyloid-like fibrillar structures in vitro. The fibrillogenesis of YB-1, as in the case of bacterial CspA [185], is mediated by its CSD domain with a β-barrel structure. It is assumed that the aggregation of YB-1 in solutions with high ionic strength is caused by the disruption of the interaction between the C-terminal region of the protein and its CSD domain (which stabilizes the β-barrel structure), and by partial denaturation of this unstable CSD domain [209]. The reversibility of the assembly of amyloid-like fibrils of YB-1 suggests the possible physiological significance of its fibrillogenesis. Moreover, the interaction between the CSD domain and the C-terminal region of the protein—and, hence, the fibrillogenesis of YB-1—can be regulated by changing the charge of this region. Furthermore, partial cleavage of the C-terminal region of YB-1 completely inhibits protein fibrillogenesis, while cleavage, conversely, stimulates its fibrillogenesis under physiological conditions [209]. It should be noted that various fragments of YB-1 are found in the cell nucleus and human blood [210,211]. The ability to form amyloid fibrils in vivo by truncated forms of YB-1 remains to be verified.

Bovine β-lactoglobulin forms two types of aggregates in vitro: (1) spherical particles (spherulites [212]) at pH values near its isoelectric point, and (2) amyloid-like fibrils at pH levels far from the isoelectric point [213], or under prolonged heating [214]. The characteristics of the obtained β-lactoglobulin aggregates significantly depend not only on pH and temperature, but also on some other factors, including selective methionine oxidation [214]. Notably, there is no direct observation of amyloid-like fibrils in milk [215]. The formation of amyloid-like fibrils by β-lactoglobulin under low-pH conditions is caused by its partial acidic hydrolysis and fibrillogenesis of the resulting peptides, as is well known for other food-derived proteins, such as casein variants and legume seed protein fractions [215].

Thus, even though proteins with a β-barrel topology are characterized by higher structural stability compared to globular α-helical proteins, and correctness of their folding and degradation is controlled by specific cellular machinery [129], some of these proteins can accumulate in partially folded states. In such states, monomers of the β-barrel proteins can interact with one another to form amyloid fibrils. Conditions initiating the fibrillogenesis of β-barrel proteins in vitro [18,186,216] suggest that the aggregation of these proteins in vivo can be promoted by some stressful external influences destabilizing the native protein structure, as well as specific cellular cofactors. Additionally, the conditions of macromolecular crowding—i.e., limited available space due to the high total concentration of macromolecules inside the cell—in some cases, are likely to stimulate the aggregation of β-barrel proteins [18]. Acceleration of fibrillogenesis in crowded environments was previously shown for a wide range of proteins [217,218]. In particular, the macromolecular crowding enhances fibril formation by the pathological human SOD1 mutant A4V [219], which is the most common familial ALS mutation in North America, and has a particularly short disease duration [220]. Observed crowding effects are at least in part due to the excluded volume-driven formation of a partially folded conformation of the proteins, which could be highly amyloidogenic [217]. Additionally, the increased viscosity and crowding-induced rearrangement of the hydrogen-bond network of water can play an important role in fibrillogenesis [218].

Every year, more and more data are accumulated on the amyloidogenic properties of proteins with a β-barrel structure predicted as their native one. Moreover, while for some of them the ability to form amyloid fibrils is currently predicted by proteomic screenings [221] or in silico methods [222,223], for others, experimental confirmation of the formation of bona fide amyloids on their basis has already been obtained in vitro and, more rarely, in vivo. Functional amyloids formed by β-barrel proteins in vivo have recently been identified in eukaryotes (plants) [18] and prokaryotes (bacteria) [62,63,64], where they are involved in nutrient storage and, probably, supra-organismal interactions. On the other hand, aberrant aggregating forms of β-barrel proteins have been found to be involved in the development of incurable diseases, such as ALS.

## 5. Interrelation between Amyloid Pathogenesis and β-Barrel Formation

**Figure 3:**
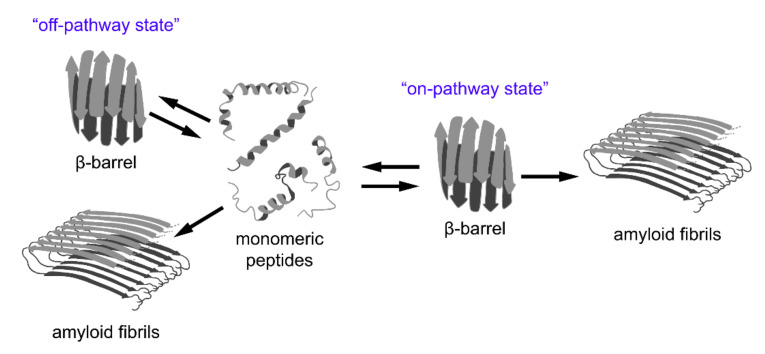
A relationship between β-barrel formation and amyloid fibril formation. According to recent observations, short peptides with different structures may form stable oligomers with β-barrel topology (so-called “cylindrins”). This state can be intermediate on the pathway of fibrillogenesis (“on-pathway state”), or can form as a result of an alternative assembly of partially unfolded monomers (“off-pathway state”). The β-barrel oligomers are considered to be highly toxic species involved in the pathogenesis of various diseases and, therefore, could represent promising targets for their treatment.

In addition to the aforementioned examples of amyloid formation by proteins with a β-barrel structure, the results of various recent studies imply that the formation of β-barrel oligomers might represent an important common intermediate step of “on-pathway” amyloid formation by different peptides and proteins (Figure 3). Alternatively, β-barrel oligomers can occur as a result of “off-pathway” amyloidogenesis but, in all cases, they are cytotoxic. In this section we review data regarding these hypotheses.

### 5.1. β-Barrel Formation as the ”Off-Pathway” of Fibrillogenesis

Using a segment of 11 amino acid residues of the slowly aggregating amyloidogenic [224] protein αB-crystallin, the possibility of forming a stable oligomer from six peptides stacked in the form of a β-barrel was shown via X-ray crystallography [225]. It turned out that αB-crystallin in oligomeric form was cytotoxic [225]. The structural transformations of the so-called “cylindrin” (toxic oligomeric β-barrel formed by extended antiparallel peptide chains) during the transition to the fibrillar state were analyzed. It was revealed that the barrel, expanded into a β-sheet, had each antiparallel pair of its β-strands not coincident with the neighboring pair by six amino acid residues. However, the β-strands in amyloid fibrils, with rare exceptions [226,227], are ”in-register” [228]—that is, the β-strands in one β-sheet must be aligned in relation to one another. This means that a β-barrel unfolded into a β-sheet will not be an “in-register” structure capable of interacting with an identical β-sheet to form an amyloid fiber core. It was shown that the transition from cylindrin to steric “zipper” occurs only after the rupture of hydrogen bonds and the reassembly of the β-strands to adopt the “in-register” structure. The results of molecular dynamics calculations indicate the significantly higher stability of fibril models than β-barrels (a difference of more than 10 kcal/mol in the free energy values was found [229]). A high free energy barrier between the β-barrel and the antiparallel fibrillar structure was also detected. This indicates that fibrils can be formed from monomers, bypassing cylindrin-like oligomeric states [229,230]. Thus, the oligomeric state with a β-barrel-type structure is probably not involved in the formation of amyloid fibrils, but is formed as a result of an alternative assembly of partially denatured molecules (“off-pathway state”). These data are consistent with the results of work on modelling the interaction of the complementary peptides CATCH (+) and CATCH (−) with opposite charges, where the formation of a β-barrel is considered to be one of the possible pathways of oligomerization, in addition to the formation of amyloid fibrils [231]. This work notes that prefibrillar oligomers are less stable than β-barrel oligomers due to a smaller number of hydrogen bonds and hydrophobic contacts.

### 5.2. β-Barrels as a Universal Intermediate State in Fibrillogenesis

In recent years, an increasing number of works have provided convincing evidence of β-barrels being a universal intermediate state that is formed during fibrillogenesis. This statement has been confirmed for peptides of various lengths with the use of a wide range of multidisciplinary approaches.

When analyzing the fibrillogenesis of seven different peptides, including those toxic (fragments of human islet amyloid polypeptide hIAPP19-29 and its mutant form S20G, as well as hIAPP22-28, Aβ16-22, and α-synuclein fragments 68–78 (NACore)) and nontoxic (hIAPP15-25 and its mutant form S20G) to cells, the formation of β-barrel oligomers by five toxic peptides was revealed via atomistic discrete molecular dynamics (DMD) [232]. The β-barrels formed by 6–8 of these peptides were aggregation intermediates that were transformed into multilayer β-sheets with an increasing oligomeric size. For the five peptides listed above, the final aggregates, when simulating large molecular systems, were similar to the experimentally observed protofibrils with paired β-sheets. The nontoxic peptide hIAPP15-25 and its mutant form S20G were shown to assemble first into unstructured non-compact oligomers, in which the content of β-sheets gradually increases with the growth in the size of the oligomers. β-sheet-enriched aggregates formed from hIAPP15-25 and hIAPP (S20G) 15–25 were polymorphic, and did not form paired multilayer β-sheets. This work considers β-barrels to be a universal intermediate state of proteins in the formation of amyloid fibrils and suggests that these intermediate β-barrel oligomers can exhibit cytotoxicity [232]. Similar conclusions about the link between the cytotoxic effect and the formation of β-barrel oligomers were obtained via modelling of the oligomerization of IAPP in membranes [233] and the fibrillogenesis of the cytotoxic fragment SOD_128–38_ and its nontoxic mutant forms [234].

The formation of highly toxic β-barrels during the process of fibrillogenesis has been exemplified by a large number of works on Aβ-peptides of various lengths. The results of molecular modelling showed that a hexamer from the C-terminal region of Aβ (1–42) can form a β-barrel enriched in hydrophobic residues and glycine residues [24]. The β-barrels formed from Aβ-peptide hexamers (including Aβ (24–34), Aβ (25–35), and Aβ (26–36)) were also identified using ion-mobility mass spectrometry combined with electron microscopy, atomic force microscopy, and computer simulation [235]. Interestingly, in the case of Aβ25–35, the possibility of the formation of a β-barrel by strands 6 and 8 of this peptide [236], along with high polymorphism of the β-strand arrangements in different variants of β-barrels formed by this peptide [237], were shown. The β-barrels formed by Aβ-peptides, in turn, can form larger assemblies [236,238], and finally form amyloid fibrils [24].

It should be noted that the model of the formation of one β-strand by one Aβ-peptide is not supported in all works. In particular, it was shown that Aβ (1–40) in a weakly alkaline solution at a low salt concentration formed β-barrels consisting of four Aβ-peptide molecules, the amino acid sequences L17–M35 of which folded into one β-hairpin (β-strand—turn—β-strand) [239]. The study of these oligomers using CD spectroscopy, size exclusion chromatography, and millisecond time-resolved hydrogen-exchange mass spectrometry made it possible to draw conclusions about the rapid mutual conversion between Aβ (1–40) oligomers and monomers. The tetrameric organization of β-barrels formed by Aβ (1–40) and Aβ (1–42) was confirmed by molecular modelling [240]. It was shown that in the lipid bilayer imitating the membrane of neurons, Aβ (1–40) and Aβ (1–42) formed β-barrels with an inner diameter of ~0.7 nm, consisting of two different variants of β-hairpins, which formed eight asymmetrically located antiparallel β-strands. The findings of this work indicate that the presence of amino acid residues 41 and 42 significantly increases the tendency of the peptide to form β-barrels, and also increases their stability. In continuation of this work, similar studies on the fibrillation of Aβ peptides in aqueous solution have shown that under these conditions, the β-barrel exists transiently for the Aβ (1–42) peptide, but this is less the case for the Aβ (1–40) peptide [240]. Similar results on the different propensity of Aβ (1–40) and Aβ (1–42) to form β-barrels were obtained using native mass spectrometry [241], extensive replica exchange molecular dynamics simulations [242], and other physicochemical approaches [243]. Confirmed by a wide range of multidisciplinary approaches (in silico, in vitro, and in vivo), the spontaneous formation of β-barrels at the early stages of Aβ42 aggregation makes it possible to conclude that these oligomers are universal toxic intermediates involved in the pathogenesis of Alzheimer’s disease [244].

Thus, the results of recent studies indicate that, despite the diversity of the primary structures of amyloidogenic proteins, during the formation of amyloid fibrils, most of them can form a universal state with a β-barrel topology. According to various concepts, this state can either be intermediate on the pathway of fibrillogenesis (“on-pathway state”), or formed as a result of an alternative assembly of partially denatured protein and peptide molecules (“off-pathway state”). However, adherents of different concepts come to the unanimous conclusion that these β-barrel oligomers are highly toxic species involved in the pathogenesis of various diseases, and can therefore be attractive targets for their treatment.

## 6. Conclusions

Numerous studies discussed above have demonstrated the relationship between the presence of β-barrel domains in the proteins and their amyloidogenic properties (Table 1). Typically, unfolded β-barrel proteins in the solutions are prone to aggregation and exhibit high structural polymorphism (Table 2), including both disordered aggregates and amyloid fibrils. For example, aggregates of YB-1, β-lactoglobulin, vicilin and its cupin domains, HPV16 E2 protein, RopA, and RopB showed an increase in β-structure content during amyloid formation [18,47,64,186,209]. For the EBNA1 protein of the Epstein–Barr virus, a decrease in the α-helical content was demonstrated. The FTIR studies of nFGF-1 and β-lactoglobulin revealed a shift in the β-sheet band from ∼1629 cm^−1^ to ∼1625 cm^−1^ [123,245], corresponding to the presence of intermolecular β-sheets typical of amyloids [225]. In addition, CD spectroscopy demonstrated the disorganization of β-barrel conformation, with consistent formation of extended β-sheets for nFGF-1 [186]. Based on these data, we may conclude that the formation of amyloid aggregates from β-barrels includes intramolecular rearrangements with an increase in β-structure content. Furthermore, considering the aforementioned data, amyloid formation is a typical variant of the supramolecular organization of unfolded β-barrel domain proteins in solutions in the absence of specific folding machinery. Other mechanisms involving the possibility of amyloid formation from the mature β-barrels remain to be investigated.

Thus, the accumulated data allow us to make assumptions about the structural changes that occur in β-barrel proteins during fibrillogenesis. The transformation of a β-barrel structure of a protein into a fibrillar structure should be facilitated by successive conformational transitions [18,47,64,186,209]. First, the destruction of hydrophobic contacts that stabilize the native structure of the β-barrel should occur, leading to the formation and accumulation of a partially folded intermediate state with extended β-sheets and loosely packed side chains [186]. This is followed by the rearrangement and annealing of the extended β-sheet elements via intermolecular hydrogen bond formation. In addition, the enhanced solvent exposure of the nonpolar side chains in the non-native intermediate protein state appears to provide a conducive environment for the condensation of the polypeptide chains [186].

The analysis of mature amyloid aggregates formed from β-barrel proteins (Table 1) has shown that they have a typical fibrillar structure [18,47,48,64,185,203,209], are resistant to treatment with ionic detergents and proteases [18,47,48,64], interact with amyloid-specific probes such as ThT, CR, and 8-(anilino)-1-naphthalenesulfonate (ANS) (which leads to a significant increase in the fluorescence quantum yield and fluorescence lifetime, as well as to a shift in the absorption and fluorescence excitation spectra of these dyes) [18,47,48,64,203], are characterized by green birefringence upon CR staining [18,64,209], have a high content of the β-sheet structure [47,48,64], and have two scattering diffraction signals characteristic of the cross-β structure [18,209] (Table 1). Thus, as a result of fibrillogenesis of the majority of studied proteins with a β-barrel structure, bona fide amyloid fibrils are formed, the properties of which are similar to those of amyloid fibrils from other amyloidogenic proteins.

Based on these data, we may propose that at least some of β-barrel proteins could be bistructural and, hence, bifunctional in vivo, being capable of the formation of (1) monomeric or oligomeric β-barrels, and (2) amyloid fibrils. The formation of amyloids in vivo under native conditions has been demonstrated for several β-barrel proteins of prokaryotes and eukaryotes. While these proteins of prokaryotes are considered to act in vivo as transmembrane pores in the folded state, their misfolding may lead to extracellular or periplasmic amyloid formation. The extracellular amyloid fibrils formed by the β-barrel proteins of Gram-negative bacteria could participate in biofilm formation or the adhesion of a pathogen or symbiont to the host [62,63,64]. Thus, in prokaryotes, β-barrel proteins seem to participate in supra-organismal interactions in both states—monomeric/oligomeric, and amyloid—acting as the virulence factors. The mechanisms of modulation of transitions between these two states in vivo remain to be investigated, but we may hypothesize that the amyloid formation by the membrane β-barrels might occur as a result of their overproduction taking place during infection, host colonization, and/or biofilm formation [246,247], and might represent a specific molecular mechanism of virulence.

In eukaryotes, known examples of the relationships between β-barrels and amyloids are mostly associated with pathogenesis, as in the aforementioned cases of amyloid formation by SOD1 and β-barrel formation by Aβ-peptide—and, more globally, toxic β-barrel oligomer formation from various amyloidogenic peptides as a common step of their amyloidogenesis. Nevertheless, in plants, β-barrel seed storage globulins accumulate in the amyloid state to enable long-term survival of seed nutrients. In addition, these amyloids of seed globulins exhibit toxicity against fungi, and might be involved in the defense of plants from pathogens. Thus, amyloids of eukaryotic β-barrel proteins could also facilitate supra-organismal interactions.

Taken together, considering the growing number of studies demonstrating amyloid formation by β-barrel proteins of prokaryotes and eukaryotes not only in vitro, but also in vivo, we can conclude that known examples of amyloids formed by β-barrel proteins are just the “tip of the iceberg”, and amyloid formation by β-barrel proteins may represent an important molecular mechanism underlying the implementation of various biological functions in prokaryotes and eukaryotes. The ability of various β-barrel proteins to form amyloids, as well as β-barrel intermediate formation by various proteins during amyloidogenesis, may reflect partial structural similarity and a close relationship between these two widespread types of β-folds.

**Table 1:** Amyloid properties of β-barrel proteins discussed in this review.

Amyloid Properties	Methods *	Protein
Formation of fibrillar structures	TEM or AFM	Vicilin [18], cupin-1.1 [18], cupin-1.2 [18], HPV16 E2 [47], RopA [64], RopB [64], CspA [185], SOD1 [208], YB-1 [209]
High turbidity and Rayleigh light scattering compared to monomeric proteins	Absorption and fluorescence spectroscopy	Vicilin [18], cupin-1.1 [18], cupin-1.2 [18], HPV16 E2 [47], EBNA-1 [48], RopA [64], RopB [64]
High content of β-sheets and β-turns	CD, FTIR	Vicilin [18], cupin-1.1 [18], cupin-1.2 [18], RopA [64], RopB [64], YB-1 [209], HPV16 E2 [47], EBNA-1 [48], nFGF-1 [186]
Resistance to treatment with ionic detergents and proteases	Treatment with denaturants/proteases	Vicilin [18], cupin-1.1 [18], cupin-1.2 [18], EBNA-1 [48], RopA [64], RopB [64]
Interaction with amyloid-specific fluorescent probes (ThT, CR);significant increase in the fluorescence quantum yield and fluorescence lifetime, as well as a shift in the absorption and fluorescence excitation spectra of the dyes upon incorporation into amyloid fibrils;ability to visualize aggregates in the presence of fluorescent probes using confocal microscopy	Tinctorial methods (including using spectroscopic approaches and confocal fluorescence microscopy)	Vicilin [18], cupin-1.1 [18], cupin-1.2 [18], HPV16 E2 [47], EBNA-1 [48], RopA [64], RopB [64], SOD1 [208]
Apple-green birefringence in polarized light when stained with CR	Polarized light microscopy	Vicilin [18], cupin-1.1 [18], cupin-1.2 [18], RopA [64], RopB [64], YB-1 [209]
The presence of two scattering diffraction signals indicative of the cross-β structure	XRD	Vicilin [18], cupin-1.1 [18], cupin-1.2 [18], YB-1 [209]

* TEM: transmission electron microscopy; AFM: atomic force microscopy; CD: circular dichroism spectroscopy; FTIR: Fourier-transform infrared spectroscopy; ThT: Thioflavin T; CR: Congo red; XDR: X-ray diffraction.

**Table 2:** Changes in the secondary structure of β-barrel proteins during amyloid formation.

Protein or Peptide	Structure of Monomer	Polymorphism	Secondary Structure Changes *	Method(s) by Which Changes in the Secondary Structure Were Detected *	Ref.
RopA	Predicted β-barrel structure	Mostly unstructured aggregates with the admixture of more ordered fibril-like structures	Before aggregation, more than 40% of β-structures; after aggregation, 42% of β-structures	CD	[64]
		Amyloid fibrils	Before aggregation, more than 40% of β-structures; after aggregation, 48% of β-structures	CD	[64]
RopB	Predicted β-barrel structure	Mostly unstructured aggregates with the admixture of more ordered fibril-like structures	Before aggregation, more than 30% of β-structures; after aggregation, 38% of β-structures	CD	[64]
		Amyloid fibrils	Before aggregation, more than 30% of β-structures; after aggregation, 44% of β-structures	CD	[64]
β-Lactoglobulin	162 amino acid residues that fold up into an 8-stranded, antiparallel β-barrel with a 3-turn α-helix on the outer surface and a ninth β-strand flanking the first strand	Spherical particles	Increase in β-sheet content in the particulate form relative to the native form of the protein, as well as a shift in the β-sheet band from ∼1629 cm^−1^ to ∼1625 cm^−1^	FTIR	[213,248]
		Fibrillar structure	Intramolecular β-sheets (Delta 1632 cm^−1^) decreased and intermolecular β-sheets (Delta 1622 cm^−1^) increased	FTIR	[123,245,249,250,251,252]
OmpA	Disordered structure in the absence of lipid bilayers	Unfolded monomer	No regular structure	CD	[61]
		Oligomeric amyloid-like state	Oligomeric form of the protein exhibited a spectrum indicative of β-sheet structure, but with a different shape and intensity than the native β-barrel spectrum	CD	[61]
YB-1	β-structure and random coil	Fibrillar structure	Increase in the content of β-structures in comparison with monomeric protein	CD	[209]
		Globular particles	CD spectra typical for unfolded proteins	CD	[209]
Vicilin (full-length)	α-helix, β-structure, and random coil	Fibrillar structure containing a fraction of less structured aggregates	Before aggregation, ~39% of β-structures; after aggregation, 41% of β-structures	CD	[18]
Cupin-1.1 (the domain of vicilin)	α -helix, β-structure, and random coil	Fibrillar structure	Before aggregation, ~4% of β-structures; after aggregation, 40% of β-structures	CD	[18]
Cupin-1.2 (the domain of vicilin)	α-helix, β-structure, and random coil	Fibrillar structure	Before aggregation, ~12% of β-structures; after aggregation, 42% of β-structures	CD	[18]
Papillomavirus HPV16 E2	α-helix, β-structure, and random coil	Monomer	Two negative bands at 212 and 225 nm indicative of β-barrel structure	CD	[47]
		Granular structures and small annuli with diameters of ∼5 nm and 10 nm, respectively	No data	No data	[47]
		Amyloid-like fibrils	Increase in the content of β-structures in comparison with monomer	CD	[47]
Epstein–Barr virus EBNA1	α-helix, β-structure, and random coil	Dimer or monomer	Characteristic bands for α-helix at 208 and 222 nm	CD	[48]
		Spherical oligomers	A decrease in α-helical content	CD	[48]
nFGF-1	All β-sheet structure	Monomer	CD data: two bands at 228 nm and 205 nm indicative of β-barrel structure; FTIR data: 1618 and 1639 cm^−1^ amide I bands indicating the β-barrel structure	CD, FTIR	[186]
		Fibrillar structure	CD data: the β-barrel conformation is disorganized (the 228 nm ellipticity band disappears), resulting in the formation of extended β-sheet conformation (formation of the negative band at 218 nm); FTIR data: 1618 and 1639 cm^−1^ amide I bands disappear; new band at1625 cm^−1^ (indicating the formation of extended β-sheets) is formed	CD, FTIR	[186]

* CD: circular dichroism spectroscopy; FTIR: Fourier-transform infrared spectroscopy.
